# Exploring the Role of the Laforin/Malin Complex in Rubicon-Dependent Phagocytosis

**DOI:** 10.3390/ijms27135787

**Published:** 2026-06-26

**Authors:** Laura Baños-Carrión, Maria Adelaida García-Gimeno, Pascual Sanz

**Affiliations:** 1 Instituto de Biomedicina de Valencia, Consejo Superior de Investigaciones Científicas, Jaime Roig 11, 46010 Valencia, Spain; lbanos@ibv.csic.es; 2 Department of Biotechnology, Escuela Técnica Superior de Ingeniería Agronómica y del Medio Natural, Universitat Politécnica de València, 46022 Valencia, Spain; magar27m@btc.upv.es; 3 Centro de Investigación Biomédica en Red de Enfermedades Raras (CIBERER), Instituto de Salud Carlos III, 46010 Valencia, Spain

**Keywords:** Lafora disease, Malin, Rubicon, ubiquitination, LAP, LANDO, astrocytes, neuroinflammation

## Abstract

Lafora disease (LD) is a fatal neurodegenerative disorder caused by mutations in the *EPM2A* or *EPM2B/NHLRC1* genes, encoding Laforin and Malin, respectively. While the Laforin/Malin E3-ubiquitin ligase complex is a known regulator of canonical autophagy and glycogen metabolism, its role in non-canonical autophagy pathways remains unexplored. Given that neuroinflammation is a hallmark of LD, we investigated the relationship between the Laforin/Malin complex and Rubicon, a critical regulator of LC3-associated phagocytosis (LAP) and LC3-associated endocytosis (LANDO). In this work, we identify Rubicon as a novel substrate and binding partner of the Laforin/Malin complex. Co-immunoprecipitation and confocal microscopy assays in HEK293 and U2OS cells demonstrated that Malin physically interacts with Rubicon, promoting its K63-linked polyubiquitination. This post-translational modification adds another layer of control to the regulation of Rubicon in specific cellular contexts. To determine the functional relevance of this interaction in LD, we assessed LAP and LANDO in primary astrocytes from Malin-deficient mice. Using flow cytometry, we quantified the engulfment and degradation of Zymosan particles and microglial debris (LAP), as well as EGF receptor internalization (LANDO). Surprisingly, no significant functional impairments were observed in Malin-deficient astrocytes compared to WT controls. These findings suggest that while the Laforin/Malin complex regulates Rubicon via K63-linked ubiquitination, redundant signaling nodes may preserve non-canonical autophagy output in Malin-deficient astrocytes.

## 1. Introduction

Lafora disease (LD, OMIM#254780, ORPHA501) is a rare and fatal progressive myoclonus epilepsy characterized by the accumulation of insoluble, aberrantly branched polyglucosan aggregates, known as Lafora bodies (LBs), in neurons and glial cells [1,2,3]. LD typically manifests in childhood or early adolescence and is characterized by generalized tonic–clonic seizures, myoclonus, absences, and visual hallucinations. The disease becomes more severe with time, with the appearance of myoclonic episodes and rapid progressive neurodegeneration, leading to the death of the patient around 10 years after the onset [4,5]. Mutations in either *EPM2A*, encoding the glucan phosphatase Laforin, or *EPM2B*/*NHLRC1*, encoding the E3 ubiquitin ligase Malin, underlie the disease and result in the disruption of a functional Laforin/Malin complex that orchestrates the recognition and ubiquitination of multiple substrates involved in glycogen metabolism, proteostasis and autophagy. Beyond glycogen accumulation, neuroinflammation has emerged as a prominent hallmark of LD, characterized by robust glial activation and increased expression of pro-inflammatory mediators [6]. Although the role of the Laforin/Malin complex in canonical autophagy is increasingly recognized [7,8], how Laforin and Malin influence alternative degradative pathways remains poorly understood.

**Figure 1:**
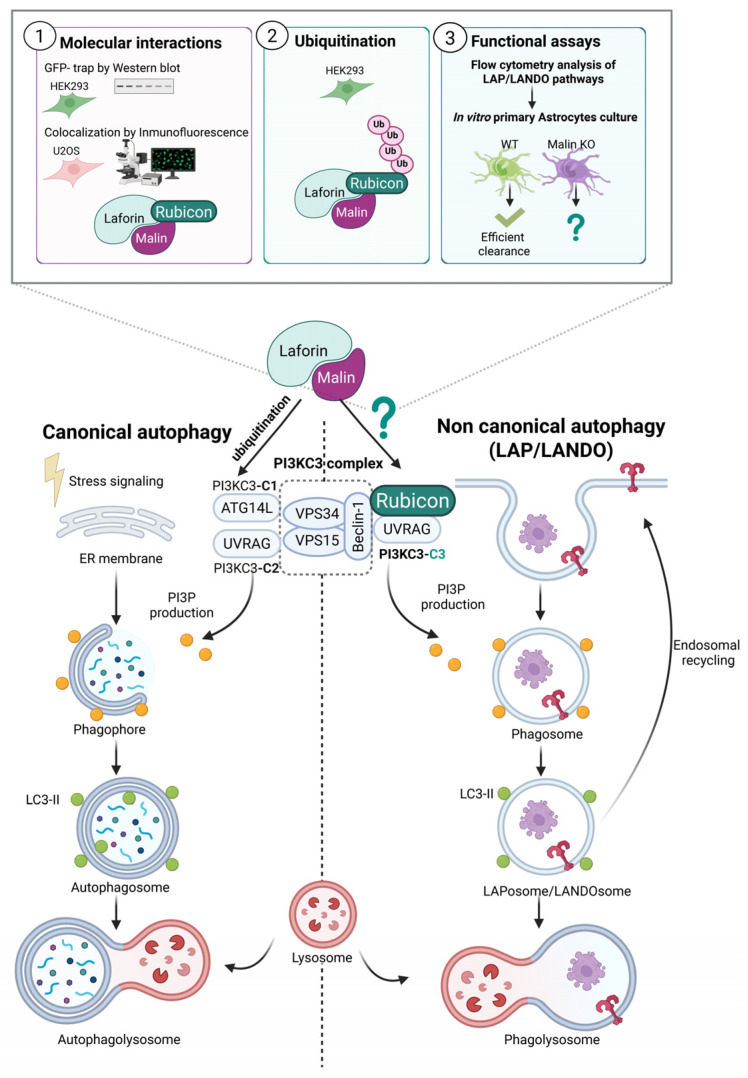
Schematic figure showing the different autophagy-related pathways [canonical autophagy and non-canonical autophagy (LAP and LANDO)]. In the upper part of the scheme, the logical flow of the study is presented. Please notice that the canonical autophagy pathway (left part of the scheme) is related to double-membrane autophagosomes. The PI3KC3 core complex, formed by VPS34, VPS15, and Beclin-1, associates with auxiliary proteins (ATG14L, to form the C1 complex, or with UVRAG, to form the C2 complex) to produce PI3P, which will attract LC3II to decorate the double membrane on both the cytosolic and the luminal faces. The Laforin/Malin complex ubiquitinates components of the PI3KC3-C1 and C2 complexes, modulating their activities [7]. In the non-canonical autophagy pathway (LAP and LANDO; right part of the scheme), structures related to these pathways contain single-membrane compartments. In this case, the PI3KC3-C3 complex (marked in green) containing Rubicon produces PI3P, which will attract LC3II only to the cytosolic face of these compartments. Finally, autophagosomes, LAPosomes, and LANDOsomes will fuse with lysosomes, where their respective cargos will be degraded. Alternatively, LAPosomes and LANDOsomes can be recycled to the plasma membrane. Created in BioRender. Predoc, U. (2026) https://BioRender.com/8llykt3, accessed on the 22 June 2026.

Since non-canonical autophagy pathways, including LC3-associated phagocytosis (LAP) and LC3-associated endocytosis (LANDO), have emerged as central regulators of innate immune signaling and extracellular cargo clearance in the brain [9,10], we analyzed the possible role of the Laforin/Malin complex in these pathways (Figure 1). A key regulator of these pathways is Rubicon (run domain Beclin-1-interacting and cysteine-rich domain-containing protein), a class III phosphatidylinositol 3-kinase (PI3KC3)-interacting protein that regulates phosphatidyl-inositol-3 phosphate (PI3P) production and LC3 lipidation at single-membrane compartments. Alterations in Rubicon function have been implicated in neuroinflammation and neurodegeneration [11,12], raising the possibility that defective Rubicon regulation may contribute to the inflammatory component of Lafora disease.

Here, we identify Rubicon as a previously unrecognized substrate and binding partner of the Laforin/Malin complex. We show that Malin physically associates with Rubicon within the Laforin/Malin complex and promotes its polyubiquitination through K63-linked ubiquitins, a post-translational modification typically associated with the modulation of protein activity and protein-protein interactions rather than degradation. As astrocytes play a main role in Lafora disease pathophysiology [6], we analyzed the possible role of this modification in primary cultures of astrocytes from *Epm2b-/-* and control mice (Figure 1). However, we did not detect functional consequences on Rubicon-dependent non-canonical autophagy pathways in Malin-deficient astrocytes, suggesting that compensatory mechanisms may operate to preserve pathway output in these cells.

## 2. Results

### 2.1. Rubicon Interacts Physically with the Laforin/Malin Complex

**Figure 2:**
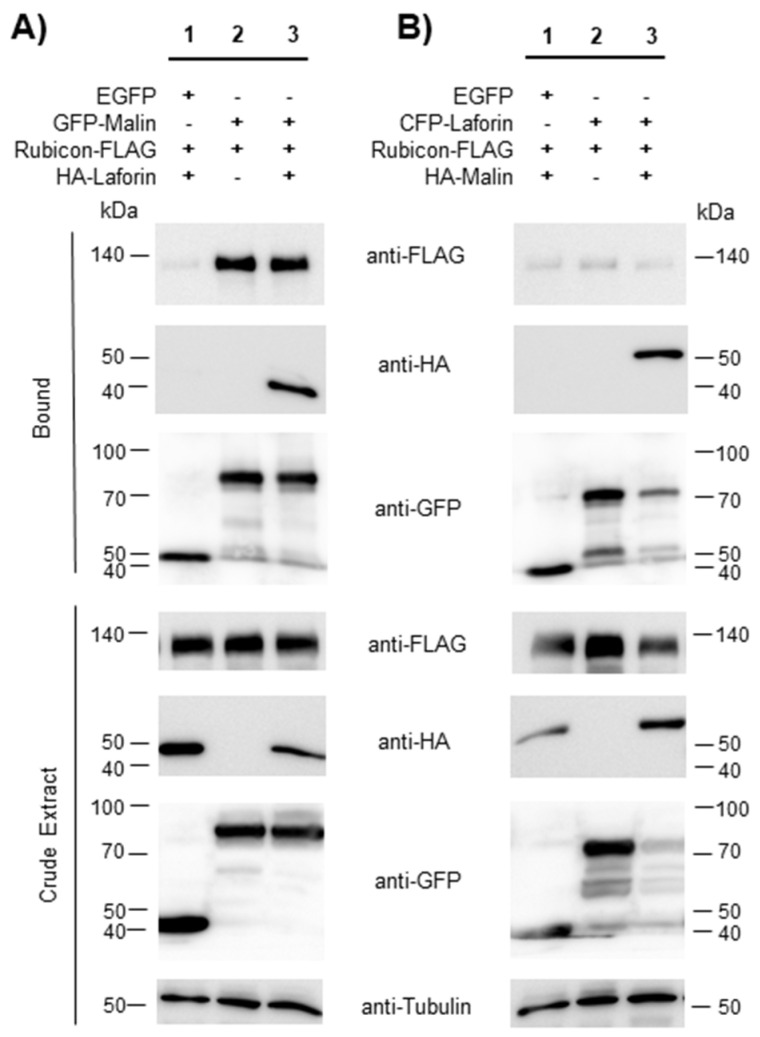
The Laforin/Malin complex interacts physically with Rubicon. (**A**) GFP-Malin pulls down Rubicon. HEK293 cells were co-transfected with the indicated combination of plasmids expressing Rubicon-FLAG, HA-Laforin, EGFP (empty), and GFP-Malin. Cells were lysed, and 1.5 mg of proteins were subjected to GFP-trap affinity purification. After washing, beads were boiled in loading buffer and the purified proteins were analyzed by SDS PAGE and Western blot using anti-GFP, anti-FLAG, anti-HA and anti-Tubulin antibodies, as indicated adjacent to the corresponding panels. Bound: proteins retained on the resin; Cell Extract: 50 μg of protein were loaded for the total fraction. (**B**) CFP-Laforin does not pull down Rubicon. HEK293 cells were co-transfected with the indicated combination of plasmids expressing Rubicon-FLAG, HA-Malin, EGFP (empty), and CFP-Laforin. Extracts were analyzed as in (**A**). Representative blots of three independent experiments are shown.

To assess whether Rubicon interacts physically with components of the Laforin/Malin complex, co-immunoprecipitation assays were performed in HEK293 cells using GFP-Trap–based affinity purification. Cells were transfected with various combinations of plasmids encoding Rubicon-FLAG, GFP-Malin, and CFP-Laforin, and the presence of interacting proteins was analyzed by immunoblotting (Figure 2). When GFP-Malin was immunoprecipitated, Rubicon co-precipitated both in the absence and presence of Laforin, indicating a direct interaction between Rubicon and Malin that does not require Laforin (Figure 2A, upper panel, lanes 2 and 3). Conversely, when CFP-Laforin was immunoprecipitated, Rubicon was not detected in the pull-down (Figure 2B, upper panel), although Malin was successfully co-precipitated, confirming the expected interaction within the Laforin/Malin complex (Figure 2B, middle panel, lane 3). These results suggest that Rubicon interacts specifically with Malin, that this interaction is preserved when Malin is part of the Laforin/Malin complex, and that the presence of Laforin does not modify this association.

Negative controls using EGFP alone did not show any binding to Malin, Laforin, or Rubicon, confirming the specificity of the observed interactions. Proper expression of all constructs was verified by Western blot analysis of whole-cell lysates (Figure 2A,B, crude extract, lower panels).

### 2.2. Rubicon Colocalizes with Malin and Its Expression Induces a Shift in the Subcellular Localization Pattern of Malin

**Figure 3:**
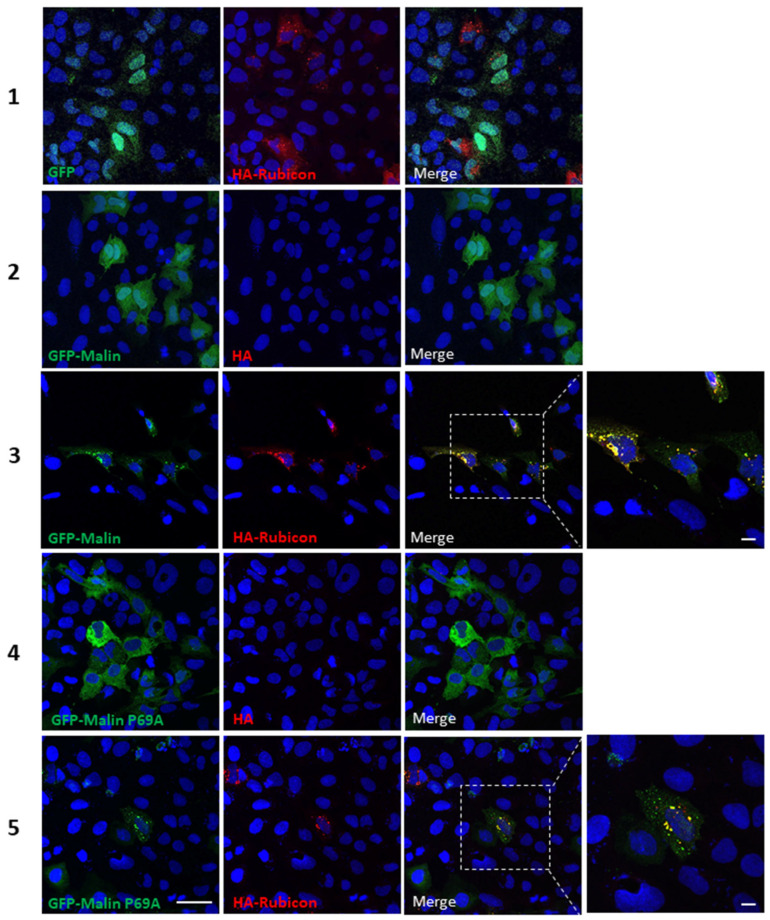
Rubicon colocalizes with both wild-type and pathological P69A Malin. Representative confocal microscopy images of U2OS cells expressing the indicated plasmid combinations. Subcellular localization was determined by direct fluorescence for GFP-tagged proteins (green) and immunofluorescence for HA-tagged Rubicon (Alexa-Fluor 633; red) as described in Materials and Methods. Nuclei were counterstained with DAPI (blue). Merge panels show the overlap of fluorescence signals; yellow indicates colocalization. Scale bars: 50 μm in main panels and 10 μm in the magnified insets. Representative images of three independent experiments are shown.

To further confirm the interaction between Rubicon and Malin, confocal immunofluorescence assays were conducted in U2OS cells (Figure 3). When GFP-Malin was expressed alone, it showed both a diffuse cytoplasmic and a nuclear distribution, as previously reported [10] (Figure 3, panel 2). The expression of Rubicon alone revealed its localization in perinuclear, vesicle-like structures (Figure 3, panel 1). However, co-expression of GFP-Malin with Rubicon resulted in a marked redistribution of Malin, which became excluded from the nucleus and accumulated in perinuclear cytoplasmic puncta that colocalized with Rubicon-positive structures (Figure 3, panel 3).

Interestingly, when we used the catalytically inactive mutant GFP-Malin-P69A, carrying the most prevalent mutation in the *EPM2B* gene, it colocalized with Rubicon in these cytoplasmic structures (Figure 3, panel 5), indicating that the interaction is preserved despite the loss of Malin’s enzymatic activity. Notably, GFP-Malin-P69A displayed an altered distribution compared to the wild-type protein, with reduced nuclear presence and a more punctate cytoplasmic pattern (Figure 3, panel 4 vs. panel 2).

These findings support a specific interaction between Rubicon and Malin, independent of Malin’s catalytic activity, and suggest that Rubicon influences the subcellular localization of both wild-type and mutant forms of Malin.

### 2.3. Rubicon Colocalizes with Laforin and Promotes a Change in Its Intracellular Distribution

**Figure 4:**
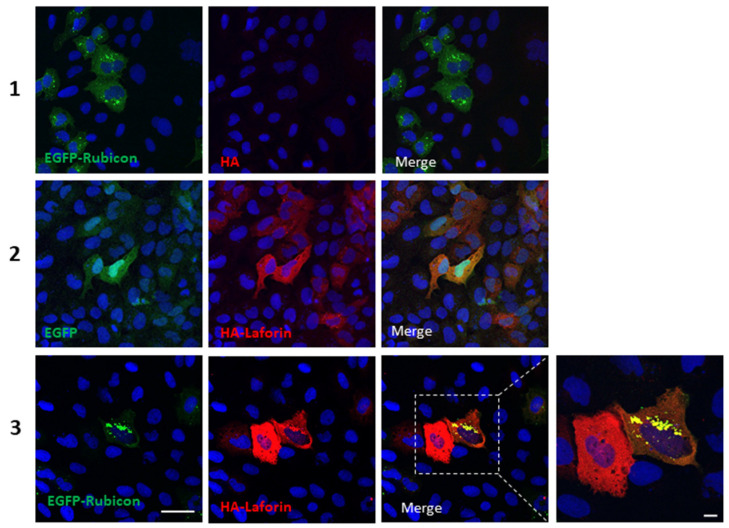
Rubicon colocalizes with Laforin. Representative confocal microscopy images of U2OS cells expressing the indicated plasmid combinations. Subcellular localization was determined by direct fluorescence for EGFP-tagged proteins (green) and immunofluorescence for HA-tagged Laforin (Alexa-Fluor 633; red), as described in Materials and Methods. Nuclei were counterstained with DAPI (blue). Merge panels show the overlap of fluorescence signals; yellow indicates colocalization. Scale bars: 50 μm in main panels and 10 μm in the magnified insets. Representative images of three independent experiments are shown.

Similar results were observed with Laforin (Figure 4). When expressed alone, HA-Laforin displayed a uniform and diffuse cytoplasmic distribution (Figure 4, panel 2), as previously reported [13,14]. However, co-expression with EGFP-Rubicon led to a marked redistribution of Laforin into punctate cytoplasmic structures that colocalized with Rubicon (Figure 4, panel 3; of note, Laforin-expressing cells that did not express Rubicon showed a cytoplasmic distribution), suggesting a physical interaction of Rubicon and Laforin, and a Rubicon-induced relocalization of Laforin, similar to that observed with Malin.

### 2.4. Rubicon Interacts Physically with the Laforin/Malin Complex and Promotes Changes in the Subcellular Localization of Both Proteins

**Figure 5:**
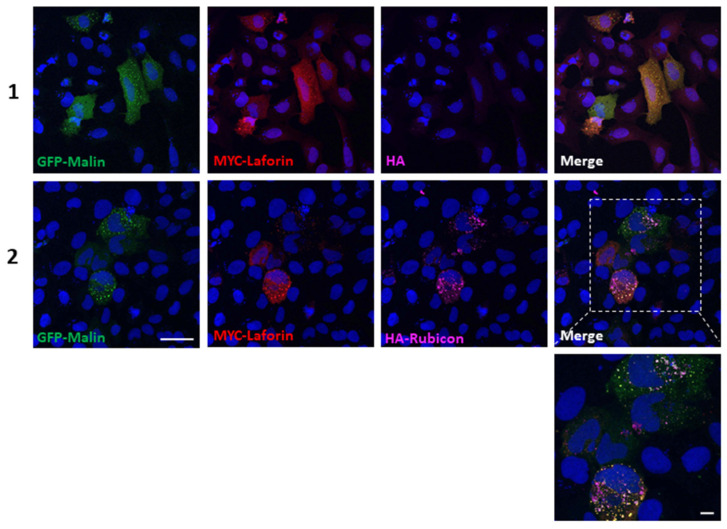
The Laforin/Malin complex colocalizes with Rubicon. Representative confocal microscopy images of U2OS cells co-expressing the indicated plasmid combinations. Subcellular localization was determined by direct fluorescence for GFP-Malin (green) and immunofluorescence for MYC-Laforin (Alexa-Fluor 568; red) and HA-Rubicon (Alexa-Fluor 633; far-red/magenta), as described in Materials and Methods. Nuclei were counterstained with DAPI (blue). Merge panels show the overlap of all fluorescence signals; colocalization of the three components is indicated by the composite signal in the magnified insets (bottom). Scale bars: 50 μm in main panels and 10 μm in the magnified insets. Representative images of three independent experiments are shown.

Finally, we examined whether Rubicon associates with the full Laforin/Malin complex. When Laforin and Malin were co-expressed in the absence of Rubicon, both proteins showed colocalization in the cytoplasm (Figure 5, panel 1). Upon co-expression with Rubicon, Laforin and Malin colocalized with this protein in cytoplasmic granular structures (Figure 5, panel 2). These results support that Rubicon is found in close proximity to the functional Laforin/Malin complex within the cell and that its presence influences the cytoplasmic distribution of both components.

### 2.5. The Functional Laforin/Malin Complex Mediates the Polyubiquitination of Rubicon by Attaching K63-Linked Ubiquitin Chains

**Figure 6:**
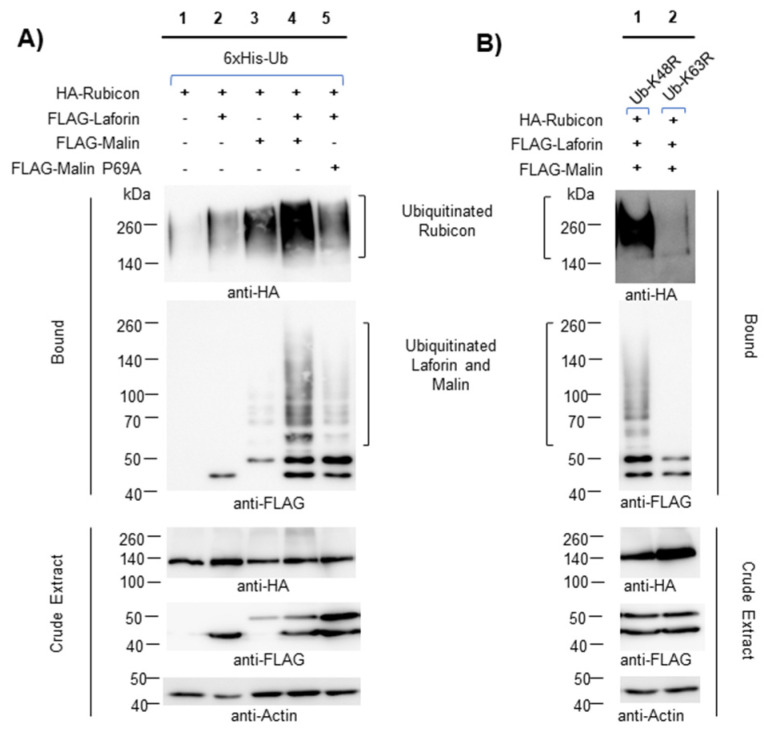
The functional Laforin/Malin complex promotes K63-linked polyubiquitination of RUBICON. (**A**) Analysis of Rubicon ubiquitination mediated by WT or catalytically inactive (P69A) Malin. HEK293 cells were co-transfected with the indicated plasmid combinations. Cells were lysed, and 1.5 mg of total protein was subjected to immobilized metal affinity chromatography (IMAC) using cobalt resin, as described in Materials and Methods. Proteins present in the bound fraction (Bound: proteins retained in the metal affinity resin) or in the crude cell extract (50 μg) were analyzed by Western blotting using the indicated antibodies. (**B**) Topology of the ubiquitination reaction. Ubiquitination reactions were performed as in (**A**) using modified forms of ubiquitin that carried K48R or K63R mutations, which prevent the formation of K48- or K63-linked chains, respectively. Representative blots of three independent experiments are shown.

To evaluate the functional relevance of the interaction between Rubicon and the Laforin/Malin complex, in vivo ubiquitination assays were performed in HEK293 cells. When Rubicon was co-expressed with both Laforin and Malin, a clear polyubiquitination signal was observed, indicating that Rubicon is a substrate of the functional complex (Figure 6A, upper panel, lane 4).

This ubiquitination was markedly reduced in the presence of the catalytically inactive mutant Malin-P69A (Figure 6A, upper panel, lane 5), demonstrating that an active Laforin/Malin complex is required for efficient modification of Rubicon. The presence of Laforin favored the ubiquitination of Rubicon; in its absence, the Malin-dependent ubiquitination of Rubicon was reduced (Figure 6A, upper panel, lane 3). Laforin promoted a minor extent of Rubicon ubiquitination (Figure 6A, upper panel, lane 2).

To determine the topology of the polyubiquitin chains involved, we employed mutant ubiquitin constructs in which either lysine 48 or lysine 63 in the ubiquitin was replaced with arginine (K48R and K63R, respectively), thereby preventing chain formation at those positions. Rubicon polyubiquitination was completely abolished in cells expressing K63R-ubiquitin (Figure 6B, upper panel, lane 2), while K48R-ubiquitin had no effect on the ubiquitination pattern compared to wild-type ubiquitin (compare Figure 6B, upper panel, lane 1 with Figure 6A, upper panel, lane 4). These findings indicate that Rubicon undergoes K63-linked polyubiquitination mediated by the Laforin/Malin complex.

The correct activity of the Laforin/Malin complex was assessed by checking its auto-ubiquitination (Figure 6A,B, middle panels), and protein expression in all conditions was verified by immunoblotting of input lysates (Figure 6A,B, crude extract).

### 2.6. Analysis of the LAP Pathway by Zymosan and Microglial Debris Uptake in Malin KO Astrocytes

**Figure 7:**
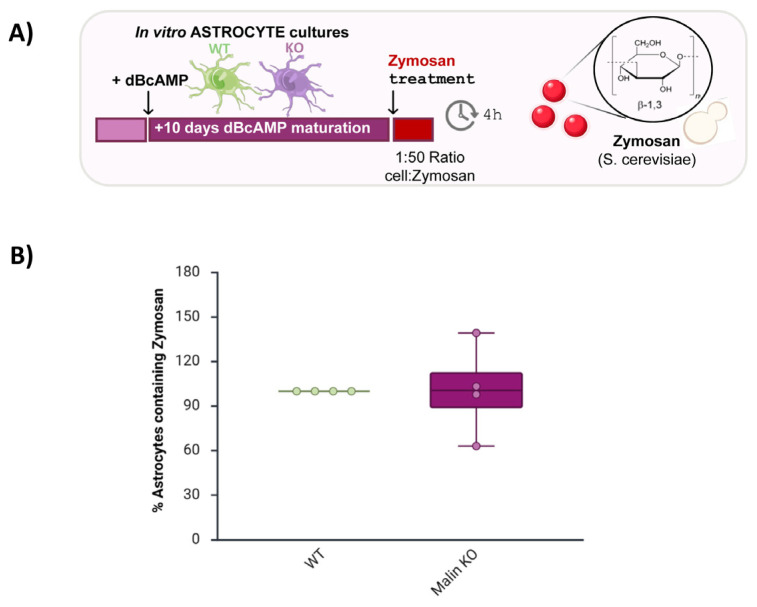
Malin deficiency does not alter Zymosan-induced LC3-associated phagocytosis (LAP) in primary astrocytes. (**A**) Schematic representation of the experimental workflow. Primary astrocytes derived from WT and Malin KO mice were treated with fluorescently labeled Zymosan particles for 4 h to induce LAP. (**B**) Quantitative analysis of phagocytic capacity by flow cytometry. Data are presented as the percentage of astrocytes containing internalized Zymosan in Malin KO astrocytes with respect to WT astrocytes, considering WT as 100% in each independent experiment. Box plots show the median (center line) and the range for each sample (n = 4 independent biological replicates). No significant differences were detected between genotypes (*p* > 0.05, Mann-Whitney U Test).

Given the functional interaction of the Laforin/Malin complex with Rubicon, a key regulator of non-canonical autophagy, we then assessed whether this relationship had downstream consequences on Rubicon-dependent pathways. First, we focused on LC3-associated phagocytosis (LAP), a process critically dependent on Rubicon activity. To directly evaluate LAP functionality, we analyzed the phagocytic capacity of primary astrocyte cultures derived from Malin KO and WT mice. Mature astrocytes were incubated with fluorescently labeled Zymosan particles, a well-established LAP inducer, for 4 h. The percentage of Zymosan-positive cells was then quantified using flow cytometry (Figure 7). Intracellular internalization of Zymosan and its colocalization with the LAP marker LC3 were confirmed by immunofluorescence (see Supplementary Figure S1, pointing arrows). No significant differences were observed between genotypes in the number of Zymosan-positive cells, indicating that the overall phagocytic capacity and LAP-mediated degradation of Zymosan remained intact in Malin-deficient astrocytes under these conditions.

**Figure 8:**
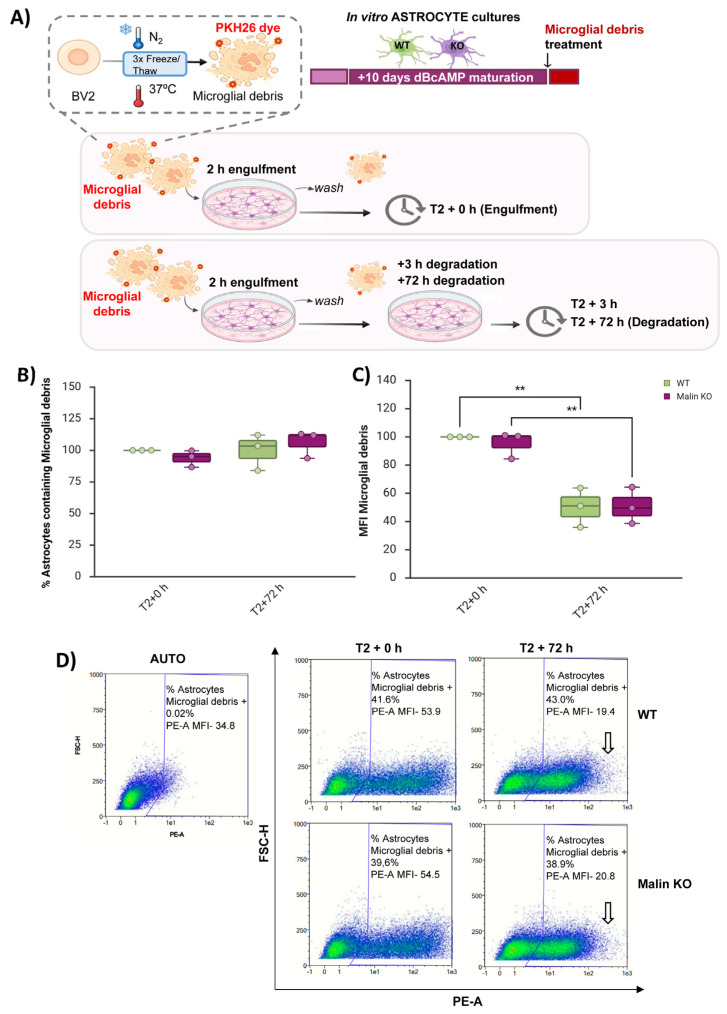
In vitro astrocytic engulfment/degradation assay using labeled microglial debris. (**A**) Diagram of the experimental workflow for the engulfment and degradation assay using fluorescently labeled microglial debris. (**B**) Quantification by flow cytometry of the percentage of Malin KO astrocytes with respect to WT astrocytes containing internalized debris after the incubation period. Box plots show the median and the range for each sample (n = 3 independent biological replicates and data was normalized considering WT T2 + 0 h as 100% in each independent experiment). (**C**) Quantitative analysis of the Mean Fluorescence Intensity (MFI) of microglial debris per cell. Box plots represent the median with the range from n = 3 independent biological replicates, and data was normalized considering WT T2 + 0 h as 100% in each independent experiment. While both WT and Malin KO astrocytes exhibit a statistically significant reduction in MFI after 72 h (** *p* < 0.01, two-way ANOVA with Bonferroni post-hoc test), indicating successful debris processing, no significant differences were observed between genotypes (*p* > 0.05, two-way ANOVA with Bonferroni post-hoc test). (**D**) Representative flow cytometry dot plots showing the proportion of astrocytes containing microglial debris and the shift in fluorescence intensity between the initial engulfment phase (0 h degradation) and the final clearance phase (72 h degradation) in both WT and Malin KO astrocytes. White arrows indicate the disappearance of fluorescence intensity in that range.

To further explore the potential impact of the Laforin/Malin complex deficiency on LAP-related processes in a more physiologically relevant setting, we assessed the ability of astrocytes to internalize and degrade labeled microglial debris. Fluorescently tagged cell remnants derived from BV2 microglial cells were used as the phagocytic substrate, and in the assay we discriminated between debris engulfment and degradation phases (Figure 8A). We first evaluated the phagocytic capacity under basal and stress conditions (LPS and heat-induced stress) (Supplementary Figure S2). While both types of stress enhanced debris engulfment, no significant differences in the proportion of cells containing microglial debris or in the Mean Fluorescence Intensity (MFI) in the cells were observed between genotypes. In the same way, no significant differences in the degradation of the debris were observed within a 3-h window in either genotype (Supplementary Figure S2). Consequently, we extended the degradation period to 72 h to ensure increased clearance. Under these conditions, WT and Malin KO astrocytes showed a comparable capacity to engulf microglial debris, as measured by the proportion of cells containing microglial debris (Figure 8B) (relative median of 100% at T2 + 0 h in WT vs. 95% at T2 + 0 h in Malin KO astrocytes, and 103% at T2 + 72 h in WT vs. 112% at T2 + 72 h in Malin KO astrocytes). In the case of the MFI (Figure 8C), there was a marked decrease in this parameter after 72 h [relative median of 100% at T2 + 0 h vs. 51% at T2 + 72 h in WT (*p* < 0.01, n = 3), and 100% at T2 + 0 h vs. 50% at T2 + 72 h in Malin KO astrocytes (*p* < 0.01, n = 3)], but no statistical differences were observed between genotypes. In Figure 8D, we present an example of the cytometry analysis performed, showing that while the percentage of debris-positive astrocytes remained stable after 72 h, there was a marked decrease in the MFI in both WT and Malin KO astrocytes. This shift is visualized in the density dot plots (PE-A vs. FSC-H), where after 72 h, the cell population remains within the positive gate but shows a clear accumulation towards lower fluorescence levels, reflecting the intracellular degradation of the internalized cargo over time (Figure 8D, white arrows).

These results indicate that under these assay conditions, the phagocytic capacity and the subsequent degradation phase of LAP are not inherently altered by the absence of a functional Laforin/Malin complex.

### 2.7. EGF Receptor Internalization via LANDO Does Not Change in Malin-Deficient Astrocytes

**Figure 9:**
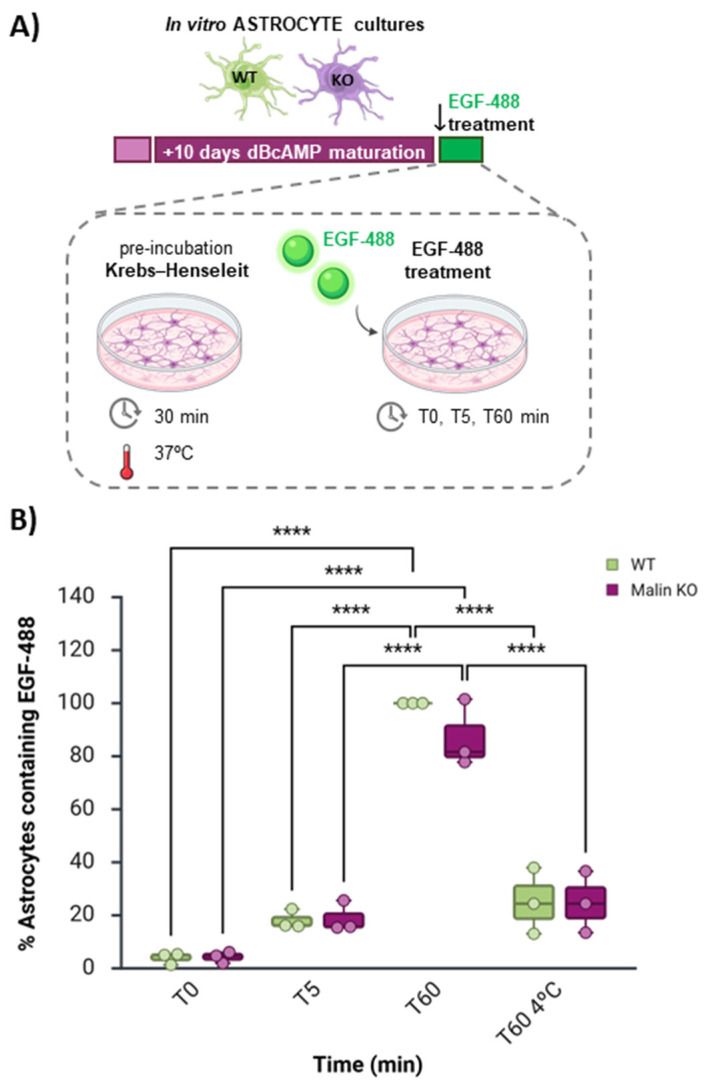
LANDO-mediated EGF receptor internalization is not impaired in Malin-deficient astrocytes. (**A**) Schematic representation of the experimental workflow for LC3-associated endocytosis (LANDO) assessment. Serum-starved primary astrocytes from WT and Malin KO mice were incubated with Alexa Fluor 488-conjugated EGF (EGF-488) to monitor receptor-mediated endocytosis. (**B**) Kinetic analysis of EGF-488 uptake, quantified by flow cytometry, over a 60-min time course. Box plots represent the median with the range from n = 3 independent biological replicates. Data was normalized to the value of WT T60 within each independent experiment. A significant time-dependent increase in EGF internalization was observed in both genotypes (**** *p* < 0.0001, two-way ANOVA with Bonferroni post-hoc), with active internalization significantly inhibited at 4 °C (negative control for the 60-min time point). No significant differences were detected between genotypes at any time point (*p* > 0.05, two-way ANOVA with Bonferroni post hoc test).

To explore the potential impact of Laforin/Malin dysfunction on the LC3-associated endocytosis (LANDO) pathway, receptor-mediated endocytosis was assessed in primary astrocytes derived from WT and Malin KO mice. Cells were incubated with Alexa Fluor 488-conjugated Epidermal Growth Factor (EGF-488), following serum starvation to promote internalization (Figure 9A). The uptake kinetics of EGF were monitored by flow cytometry across a 0–60 min time course. A negative control was included by incubating parallel samples at 4 °C to inhibit active endocytosis, confirming that the observed signal reflected true internalization events. This control was performed only for the longest incubation time.

We observed an increase in the uptake of the ligand with time (*p* < 0.0001, n = 3). However, no significant differences were detected between genotypes in the rate of EGF-488 uptake at any of the time points analyzed (Figure 9B) (relative median of 5% in WT vs. 5% in Malin KO at 0 min; 16% in WT vs. 16% in Malin KO at 5 min; 100% in WT vs. 82% in Malin KO at 60 min; and 24% in WT vs. 24% in Malin KO at 60 min and 4 °C), indicating that the LANDO pathway remains functional in Malin-deficient astrocytes.

Given the known role of Rubicon as a key regulator of non-canonical autophagy, including both LAP and LANDO, these findings suggest that the loss of Rubicon ubiquitination in the absence of a functional Laforin/Malin complex does not impair either LAP or LANDO in astrocytes under the conditions tested.

## 3. Discussion

Lafora disease (LD) is a rare autosomal recessive neurodegenerative disorder characterized by the accumulation of poorly branched, insoluble glycogen-like inclusions known as Lafora bodies (LBs). Among the cellular hallmarks of LD are alterations in proteostasis, including significant defects in autophagy. The Laforin/Malin complex, mutated in LD, has been shown to regulate components of the canonical autophagy machinery, notably through the ubiquitination of members of the PI3KC3 complex [6]. However, non-canonical autophagy pathways such as LC3-associated phagocytosis (LAP) and endocytosis (LANDO), which share upstream components with canonical autophagy but diverge functionally, remain unexplored in the context of LD. Importantly, dysregulation of these pathways has been increasingly linked to neuroinflammatory responses, another core feature of LD pathophysiology [4,5]. The present study provides novel insights into the functional relationship between the Laforin/Malin complex and Rubicon, a regulator of non-canonical autophagy, in the context of LD. Our results demonstrate a previously unreported interaction between Rubicon and the Laforin/Malin complex, both at the physical and functional levels. We report a physical interaction between Rubicon and the Laforin/Malin complex and also a colocalization of the three proteins in aggregated perinuclear structures. In addition, we report that the expression of Rubicon changes the regular subcellular localization of both Laforin and Malin towards these structures. This capacity of Rubicon to act as a molecular scaffold, sequestering its binding partners and dictating their intracellular distribution, aligns with its previously described role in the spatial regulation of the Beclin-1-UVRAG and HOPS complexes [15,16]. The Laforin/Malin complex is known to catalyze the formation of K63-linked polyubiquitin chains on several substrates [see [8,17] for review]. Consistent with this, using HEK293 cells, we show that Laforin/Malin mediates K63-linked polyubiquitination of Rubicon. Interestingly, we did not observe a decrease in Rubicon protein levels following Malin-dependent ubiquitination. These findings align with previous literature describing how K63-linked ubiquitination of autophagy regulators modulates their activity without targeting them for proteasomal degradation [17].

Rubicon has previously been reported to undergo other post-translational modifications, including phosphorylation [18], and more recently, ubiquitination by other E3 ligases such as HECTD1 [19], which targets Rubicon for proteasomal degradation. Our data position Malin as a novel E3 ligase capable of modifying Rubicon, potentially adding another layer of control on the regulation of Rubicon in specific cellular contexts. The specificity of this ubiquitination suggests that the Laforin/Malin complex could act as a fine-tuning regulator of Rubicon function, particularly in processes like LAP and LANDO, in which Rubicon has been shown to play essential roles [10,20].

Despite this functional relationship, our downstream analyses suggest that the loss of Malin does not globally impair non-canonical autophagy in astrocytes derived from LD mouse models. We assessed the ability of primary astrocytes to phagocytose Zymosan particles, a classical LAP stimulus [21], by flow cytometry and found that, under our working conditions, we did not detect differences in the phagocytic capacity of astrocytes from Malin-deficient mice with respect to WT counterparts. Alternatively, we used labeled microglial debris in the reaction, and again we did not observe genotype-dependent changes in either cargo engulfment or degradation. Furthermore, receptor-mediated endocytosis assessed via EGF-Alexa Fluor 488 internalization, a readout of LANDO functionality, also appeared unaltered in Malin-deficient astrocytes in comparison to WT. These results suggest that alternative mechanisms may exist in Malin-deficient astrocytes that preserve LAP and LANDO functionality, despite upstream post-translational modifications in Rubicon, which require further investigation. Similar redundancies have been proposed in other systems, where defects in one LAP regulator can be bypassed by alternative signaling nodes [22]. Additionally, recent findings highlight the highly context-dependent nature of Rubicon [23]. Notably, in that study, the authors observed significant changes in microglia and neurons from Rubicon-mutant mice, but found that astrocyte markers remained unaltered in these cells. This supports the idea that, in primary astrocytes, the functional consequences of an altered Laforin/Malin–Rubicon axis might be compensated by redundant pathways. This is perhaps one limitation of our study, and further investigations should be carried out in microglia and/or neurons to check the role of the Laforin/Malin complex in these specific cells.

## 4. Materials and Methods

### 4.1. Mammalian Cell Culture

Human embryonic kidney cells (HEK293) (HPA Culture Collection #851820602, Salisbury, UK) and human osteosarcoma U2OS cells (Public Health England #92022711, Salisbury, UK) were used for transfection experiments. BV2 cells (IRCCS AOU San Martino IST #ICLC ATL03001, Genova, Italy) were cultured to generate microglial debris for phagocytosis assays, as detailed in Section 4.9. All cell lines were grown in Dulbecco’s modified Eagle medium (DMEM) (Lonza, Barcelona, Spain), supplemented with 10% heat-inactivated fetal bovine serum (FBS) (Invitrogen, Madrid, Spain), 1% L-glutamine, 100 units/mL penicillin, and 100 μg/mL streptomycin in a humidified atmosphere at 37 °C and 5% (*v*/*v*) of CO_2_.

### 4.2. Primary Mouse Astrocyte Isolation and Culture

This study was carried out according to the recommendations in the Guide for the Care and Use of Laboratory Animals of the Consejo Superior de Investigaciones Cientificas (CSIC, Madrid, Spain) and approved by the Consellería de Agricultura, Medio Ambiente, Cambio Climático y Desarrollo Rural from the Generalitat Valenciana. Mouse procedures were approved by the Animal Ethics Committee of the Instituto de Biomedicina de Valencia-CSIC [Permit Number: IBV-51, 2019/VSC/PEA/0271, authorized date 30 June 2021]. All efforts were made to minimize animal suffering. Mouse primary astrocytes from control and *Epm2b-/-* mice (Malin KO) were obtained from P0 to P1 mice, as in Moreno-Estelles et al. (2023) [24]. The cells were grown in DMEM (Lonza, Barcelona, Spain) containing 20% inactivated fetal bovine serum (FBS) (Fisher Scientific, Madrid, Spain), supplemented with 1% L-glutamine, 7.5 mM glucose, 100 units/mL penicillin, and 100 μg/mL streptomycin, in a humidified atmosphere at 37 °C with 5% CO_2_. After 48 h, FBS was reduced to 10%. For the following 2 weeks, 0.25 mM dibutyryl-cAMP (dBcAMP) (D0627, Sigma-Aldrich, Madrid, Spain) was added to the cultures to favor astrocyte maturation. At the end of the maturation process, primary astrocytes were grown for a further 24 h in the same media but in the absence of dBcAMP to avoid any secondary effect deriving from the compound [25,26].

### 4.3. Immunofluorescence Analyses

The cells were fixed with 4% PFA in 1× phosphate-buffered saline (PBS) for 15 min. Plates were immersed in blocking buffer (3% BSA, 0.02% saponin in 1× PBS) and incubated overnight at 4 °C with LC3BII (#PM0369, MBL, 1:1000) diluted in blocking buffer. After three washes for 10 min in blocking buffer, the cells were incubated for one hour at room temperature with the appropriate secondary antibody diluted at 1:500 in blocking buffer, washed twice with blocking buffer and once with 1× PBS, and mounted in Fluoroshield with DAPI (F6057, Sigma, Madrid, Spain). Images were acquired with a Confocal Spectral Zeiss LSM 980 microscope (Zeiss group, Jena, Germany). All images were acquired using the 63× objective, at one unit Airy pinhole, with a resolution of 1024 × 1024 pixels and z-stacks of 0.2 μm. At least four independent samples were analyzed for each staining and condition (control and *Epm2b-/-* mice). For each staining, the percentage of the corresponding laser intensity and detector gain settings was maintained between samples.

### 4.4. Plasmid Constructs

The following plasmids were described by Sanchez-Martin et al. (2015) [7]: pFLAG-Laforin, pECFP-Laforin, pEGFP-Malin, and pFLAG-Malin. Plasmid pFLAG-Malin P69A was described in reference (Couarch et al., 2011) [27]. The Rubicon (RUBCN) coding sequence was obtained from the commercial plasmid pcDNA6/TO EGFP-RUBICON (Addgene plasmid #28022). Custom constructs were generated by standard molecular cloning techniques to produce variants with specific tags, such as EGFP or HA, as required for different experimental applications. Further details on cloning strategies are available upon request. Plasmid pCMV-6xHisUbiq was generously provided by Dr. Manuel Rodríguez (Proteomics Unit, CIC-bioGUNE, Bizkaia, Spain) and plasmids pCMV-6xHis-Ubiq-K48R and pCMV-6xHis-Ubiq-K63R were a generous gift of Dr. Ch. Blattner (Institute of Toxicology and Genetics, Karlsruhe Institute of Technology, Karlsruhe, Germany).

### 4.5. Analysis of Protein Ubiquitination

The method described in [28] was used to study the ubiquitination of Rubicon. For this purpose, HEK293 cells were transfected with the plasmids indicated in each experiment using X-treme GENE HP transfection reagent according to the manufacturer’s protocol (Roche Diagnostics, Barcelona, Spain). After 24 h of transfection, the cells were lysed using a 25-gauge needle in buffer A (6 M guanidinium-HCl, 0.1 M sodium phosphate, 0.1 M Tris-HCl pH 8.0) to inhibit the action of endogenous deubiquitinases. Protein extracts were clarified after centrifugation (12.000 rcf, 15 min) and protein concentration was measured using the Bradford technique. In total, 1.5 mg of protein was incubated with 200 μL of a TALON cobalt resin (Clontech, Barcelona, Spain) equilibrated in buffer B containing 10 mM imidazole, 6 M guanidinium-HCl, 0.1 M sodium phosphate, 0.1 M Tris-HCl pH 8.0. To purify His-tagged proteins, incubation was carried out for 2 h at room temperature on a rocking platform. Then, the resin was washed with 1 mL of buffer B and four times with buffer C (buffer B, but with 8 M urea instead of 6 M guanidinium-HCl). Bound proteins were boiled at 95 °C for 5 min in 50 μL of 2 × Laemmli’s sample buffer and analyzed by Western blotting using the appropriate antibodies. To determine the topology of the ubiquitin chains, when indicated, plasmids pCMV-6xHisUbiq-K48R and pCMV-6xHis-Ubiq-K63R were used in the assay instead of pCMV-6xHis-Ubiq wild type.

### 4.6. GFP-Trap Analysis of Protein-Protein Interactions

HEK293 cells were transfected with specific constructs of Laforin, Malin, and the protein of interest. The cells were washed twice with cold 1× PBS and scraped on ice in lysis buffer [10 mM Tris–HCl pH 7.5, 150 mM NaCl, 0.5 mM EDTA, 0.5% (*v*/*v*) Nonidet P-40, complete protease inhibitor cocktail (Roche Diagnostics, Barcelona, Spain), 1 mM PMSF, 2.5 mM NaF, 0.5 mM NaVO4, and 2.5 mM Na4P2O7]. The lysates were collected in an Eppendorf tube, and further lysis was performed using a 25-gauge needle. The cell lysates were then centrifuged at 13,000 rcf for 10 min at 4 °C. Supernatants (1.5 mg of total protein) were incubated with Chromotek GFP-trap beads (Chromotek, Planegg-Martinsried, Germany) for 1 h on a rocking platform at 4 °C. Beads were washed two times with 1 mL of lysis buffer and one time with the lysis buffer containing 300 mM NaCl. Bound proteins were boiled at 95 °C for 5 min in 30 μL of 2 × Laemmli’s sample buffer. The GFP- and CFP-fused proteins were pelleted and visualized by immunoblotting using specific antibodies. As a negative control, a construct expressing EGFP was used to confirm the specificity of the interaction.

### 4.7. Western Blot Analyses

In total, 30 μg of total protein from the soluble fraction of cell lysates were analyzed by SDS-PAGE, and proteins were transferred to PVDF membranes (Millipore, Madrid, Spain). Membranes were blocked with 5% (*w*/*v*) non-fat milk in Tris-buffered saline Tween20 buffer [TBS-T: 50 mM Tris-HCl pH 7.4, 150 mM NaCl, 0.1% (*v*/*v*) Tween20] for 1 h at room temperature and incubated overnight at 4 °C with the corresponding primary antibodies: mouse anti-HA (H9658, Sigma-Aldrich), mouse anti-MYC (M5546, Sigma-Aldrich), mouse anti-FLAG (F3165, Sigma-Aldrich, Madrid, Spain), rabbit anti-GFP (210-PS-1GFP, Inmunokontackt, Madrid, Spain). Mouse anti-Tubulin (T6199, Sigma-Aldrich, Madrid, Spain) and rabbit anti-Actin (A2066, Sigma-Aldrich, Madrid, Spain) were used as loading controls. After washing, the membranes were incubated with the corresponding HRP-conjugated secondary antibodies for 1 h at room temperature. Signals were visualized using Lumi-Light Western Blotting Substrate (Roche Applied Science, Barcelona, Spain) or ECL Prime Western Blotting Detection Reagent (GE Healthcare, Barcelona, Spain), and analyzed by chemiluminescence using the FujiLAS400 (GE Healthcare, Barcelona, Spain) image reader.

### 4.8. Zymosan Uptake Assay

For phagocytosis assays, primary astrocytes were incubated with Texas Red-conjugated Zymosan particles (Zymosan A BioParticles™, Thermo Fisher, Z-2843, Madrid, Spain) at a cell-to-particle ratio of 1:50. Particles were reconstituted and handled according to the manufacturer’s instructions, including proper resuspension and quantification prior to use. Astrocytes were incubated with the particles for 4 h at 37 °C, after which the cells were washed thoroughly with 1× PBS to remove non-internalized particles. The samples were then analyzed by fluorescence microscopy and flow cytometry.

### 4.9. In Vitro Debris Phagocytosis Assay

To generate cellular debris, BV2 microglial cells were subjected to repeated freeze–thaw cycles. Specifically, confluent BV2 cultures were collected and resuspended in 1× PBS, then subjected to three consecutive cycles of freezing in liquid nitrogen followed by thawing at 37 °C. The resulting lysate was stored at −80 °C until use. The dead microglia were labeled with PKH26 red fluorescence dye (MINI26, Sigma, St. Louis, MO, USA). The labeling protocol was adapted to the sample volume and protein concentration. Typically, for every 15 µL of debris suspension (2 µg/µL), 3 µL of PKH26 dye were mixed with 210 µL of diluent provided in the kit. Corresponding adjustments in dye and diluent volumes were made accordingly to ensure consistent staining efficiency. The debris was incubated in this mixture for 5 min at room temperature with gentle agitation. The staining reaction was quenched by the addition of 1% BSA in 1× PBS, followed by centrifugation at 300 rcf for 7 min. The labeled debris pellet was resuspended in astrocyte culture medium prior to addition to the cells.

To test whether the cell debris could be engulfed by cultured cells, the labeled debris was added to primary astrocyte cultures at a final concentration of 1 µg per 10,000 seeded astrocyte cells. To allow discrimination between engulfment and degradation phases, the astrocytes were incubated with the labeled debris for 2 h, after which the non-engulfed debris was removed by washing with 1× PBS. The astrocytes were then either fixed and processed to evaluate debris internalization or cultured for an additional 3 to 72 h to evaluate degradation dynamics. These assays were performed under basal or stress conditions, including thermal stress or LPS stimulation, as described in each experiment. Phagocytic capacity was analyzed by flow cytometry.

### 4.10. Debris Treatment Under Stress Conditions

For experiments assessing the impact of cellular stress on debris clearance, primary astrocyte cultures were subjected to two different stress paradigms during debris exposure. In the case of thermal stress, astrocytes were incubated at 42 °C for 1 h during the engulfment phase of labeled BV2-derived debris. Following this initial stress period, the cells were returned to 37 °C for an additional 3-h degradation phase, as longer exposure to heat shock conditions proved cytotoxic in preliminary tests.

For inflammatory stress conditions, debris was generated from BV2 microglial cells pre-treated with lipopolysaccharide (LPS, 1 μg/mL) for 3 h, with adenosine triphosphate (ATP, 1 mM) added during the last 45 min of the incubation without removing LPS. After the LPS/ATP stimulation, the cells were subjected to freeze–thaw cycles to generate debris as described above, and this proinflammatory debris was subsequently added to astrocyte cultures following the standard engulfment/degradation assay protocol.

### 4.11. EGF Internalization

To assess receptor-mediated endocytosis dynamics, astrocytes were pre-incubated for 30 min at 37 °C in Krebs–Henseleit buffer, following extensive washing with 1× PBS to remove any residual culture medium. Immediately after, Alexa Fluor™ 488-conjugated Epidermal Growth Factor (EGF-488, Thermo Fisher, E13345) was added to the cells at a final concentration of 2 µg/µL, and internalization was allowed for different time points (0, 5, and 60 min) at 37 °C. At each designated time point, the cells were rapidly washed with cold 1× PBS containing 0.1% BSA, centrifuged at 300 rcf for 10 min at 4 °C, and resuspended in cold 0.1% BSA in 1× PBS for subsequent flow cytometry analysis. As a negative control for endocytosis, a parallel set of astrocytes was incubated with EGF-488 under identical conditions but maintained at 4 °C to inhibit active internalization.

### 4.12. Flow Cytometry Analysis

For flow cytometry analysis, primary astrocytes were detached using 0.25% trypsin (Trypsin- EDTA 0.25%, phenol-red, Thermo Fisher, 25200056) for 3 min at 37 °C. Cells were then collected in the astrocyte medium and centrifuged at 300 rcf for 5 min at room temperature. The cell pellets were resuspended in cold 1× PBS containing 0.5% BSA and kept on ice for subsequent staining procedures. Preliminary experiments were conducted to ensure the purity and viability of the primary astrocyte cultures. To specifically identify astrocytes, the cells were incubated with ACSA-2-FITC antibody (Cat#:130-116-243, Miltenyi Biotec, Barcelona, Spain; dilution 1:50) for 10–15 min at room temperature in the dark, consistently yielding >95% pure populations (as characterized in [24]). To assess cell viability, Viobility™ 405/520 Fixable Dye (Cat#: 130-130-404, Miltenyi Biotec) was added simultaneously, following the manufacturer’s instructions. However, given the consistently high viability (>95%) and the purity of the culture, these markers were omitted in subsequent functional assays to streamline the protocol. These preliminary validations included necessary controls, such as unstained cells (autofluorescence) and Fluorescence Minus One (FMO) to define gating boundaries. After staining, the cells were washed with cold 0.5% BSA in 1× PBS and centrifuged again at 300 rcf for 5 min at 4 °C. The supernatant was discarded, and the cells were resuspended in 1× PBS 0.5% BSA prior to acquisition.

Samples were analyzed on a MACSQuant^®^ Analyzer 10 flow cytometer (Miltenyi Biotec). For each experiment, an unstained control (Auto) was used to set positivity thresholds and account for cellular autofluorescence. After exclusion of debris and doublets through FSC/SSC and FSC-A/FSC-H gating, a minimum of 10,000 events were analyzed per sample. Specific uptake was quantified as follows: (i) Zymosan Uptake (LAP): Detected as Alexa Fluor 594 signal (PE channel); (ii) Microglial Debris Clearance (LAP): Monitored via PKH26 dye-red fluorescence (PE channel); (iii) EGF Internalization (LANDO): Quantified as Alexa Fluor 488 signal (FITC channel). Data were processed using MACSQuantify 2.13.3 software. To account for inter-experimental variability, the percentage of positive cells and MFI were normalized, considering the value of the WT as 100% in each independent biological replicate.

### 4.13. Statistical Analysis

Statistical analyses were performed using Biorender.com. Data are presented as median with a range from at least three independent biological replicates. The normality of the data distribution was assessed to determine the appropriate statistical tests. For comparisons between two experimental groups (Zymosan uptake), an unpaired, non-parametric Mann–Whitney U test was employed. For experiments involving multiple time points and genotypes (microglial debris clearance and EGF internalization kinetics), a two-way ANOVA followed by Bonferroni’s post hoc test for multiple comparisons was used. Statistical significance was defined as: * *p* < 0.05, ** *p* < 0.01, *** *p* < 0.001, and **** *p* < 0.0001. In all cases, *p*-values > 0.05 were considered non-significant.

## Data Availability

The original contributions presented in this study are included in the article/Supplementary Materials. Further inquiries can be directed to the corresponding author.

## Supplementary Materials

The following supporting information can be downloaded at https://www.mdpi.com/article/10.3390/ijms27135787/s1.

## Abbreviations

The following abbreviations are used in this manuscript:

LANDO	LC3-associated endocytosis
LAP	LC3-associated phagocytosis
LD	Lafora disease
PIP3	Phosphatidyl inositol 3 phosphate
