# Supplementary material for: Exploring the Role of the Laforin/Malin Complex in Rubicon-Dependent Phagocytosis
Source: Int J Mol Sci. 2026 Jun 26;27(13):5787. doi: 10.3390/ijms27135787 (PMC13362185; doi:10.3390/ijms27135787)

Supplementary information:

Supplementary Figure S1: Zymosan internalization and its recruitment to LC3-positive phagosomes in primary astrocytes. Representative confocal microscopy images of WT and Malin KO astrocytes incubated with fluorescently labeled Zymosan particles (red) for 4 hours. Immunofluorescence analysis shows the formation of LC3-positive puncta (green) surrounding internalized Zymosan (pointing arrows), indicative of LC3-associated phagocytosis (LAP) functionality. Nuclei were counterstained with DAPI (blue). Scale bars: 20 µm.

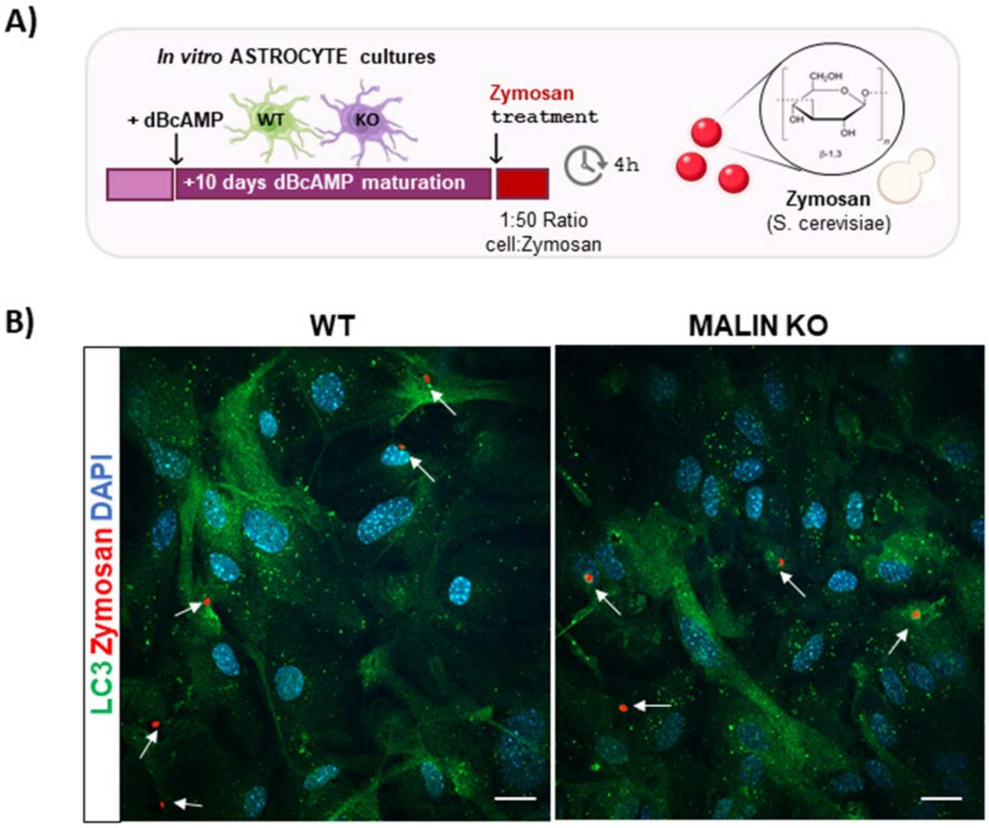

Supplementary Figure S2: Stress conditions enhance astrocytic engulfment but no degradation was observed either in WT or KO astrocytes after 3h. A) Schematic representation of the experimental workflow under basal and stress conditions (LPS-stimulation and heat shock). Astrocytes were exposed to microglial debris for 2 hours, followed by a 3-hour degradation period to assess early-phase clearance dynamics. B) Flow cytometry quantification of the percentage of debris-positive astrocytes across the different experimental conditions. Stress paradigms (LPS and thermal stress) notably enhanced the proportion of engulfing cells, but similarly in both WT and Malin KO astrocytes. C) Analysis of Mean Fluorescence Intensity (MFI) per cell at 0 and 3 hours post-engulfment. No substantial reduction in MFI was observed within this short interval under any condition, indicating that 3 hours is insufficient to detect debris degradation. These pilot observations (representative of n=1 biological replicate) justified the extension of the degradation phase to 72 hours in subsequent definitive experiments.

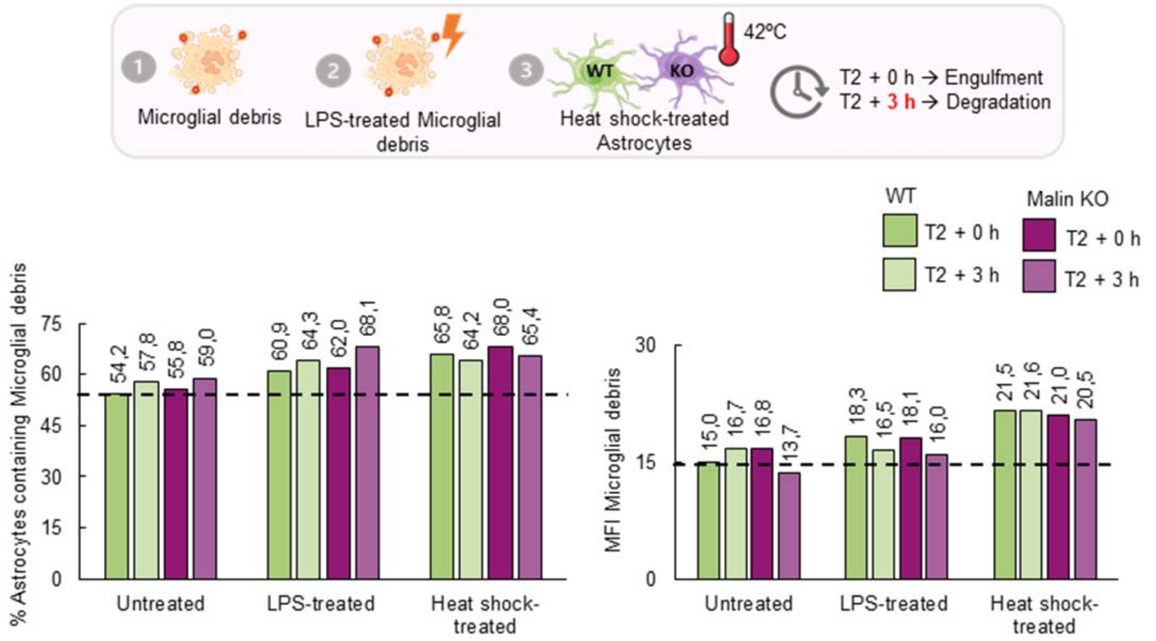

Supplement: Supplementary file 1 [file ijms-27-05787-s001.zip › ijms-4327620-supplementary.pdf]
